# Impact of Sickle Cell Awareness Day on online health information seeking in Africa using Google Trends

**DOI:** 10.1093/heapro/daad152

**Published:** 2023-12-02

**Authors:** Emma Parry, Idayat Ayinla-Jimoh, Thomas A Shepherd

**Affiliations:** School of Medicine, Keele Road, Keele University, ST5 5BG, Staffordshire, UK; School of Medicine, Keele Road, Keele University, ST5 5BG, Staffordshire, UK; School of Medicine, Keele Road, Keele University, ST5 5BG, Staffordshire, UK

**Keywords:** Sickle Cell Disease, Google Trends, online health information seeking behaviour, Africa, disease awareness

## Abstract

The United Nations Council Assembly recognized sickle cell disease (SCD) as a global public health problem due to its increasing burden, particularly in sub-Saharan Africa. To raise awareness, a resolution was adopted, designating June 19th as SCD awareness day. However, the impact of this awareness day on online health information seeking behaviour (OHISB) in African countries is not well understood, especially in Nigeria, Ghana and Uganda where SCD prevalence is high. To assess the impact, the study used Google Trends data as a measure of OHISB for SCD. The analysis covered the 60 days before the awareness day, the awareness day itself, and the 60 days afterward. Time series analysis was conducted using joinpoint regression to identify significant changes in OHISB trends. The results indicated that the impact of the Sickle Cell Awareness Day on OHISB varied across African countries and did not consistently inspire significant changes in information seeking behaviour. This suggests the need for more targeted awareness campaigns to improve public knowledge of SCD in Africa. It also highlights the importance of revising the current awareness day or creating alternative health awareness initiatives that adopt a long-term approach and address the specific health needs of the African population. Furthermore, due to limitations in using Google Trends data in some African countries with insufficient data, future research should explore other sources of internet data or conduct surveys to gain a more comprehensive understanding of the impact of the Sickle Cell Awareness Day on OHISB in Africa.

**Contribution to Health Promotion**

- Impact of the Sickle Cell Awareness Day on online health information seeking behaviour (OHISB) using Google Trends data was not consistent across sub-Saharan African countries and generally the impact was short lived.
- Kenya and Nigeria saw the most significant increase in OHISB following the awareness day but soon returned to previously observed levels.
- Low levels of internet penetrance in certain African regions may limit the impact of health awareness days on OHISB, reinforcing the importance of utilizing different methods (such as radio, television and local media) to disseminate health information.

## INTRODUCTION

Sickle Cell Disease (SCD) is a group of blood disorders that leads to a change in the shape of erythrocytes into a sickle shape (Rees *et al*., 2010). It is one of the most common inherited conditions, leading to recurrent sickling and anaemia from the breakdown of red blood cells (Ware *et al*., 2017). The resultant chronic organ damage causes high morbidity and early mortality (Ware *et al*., 2017). With SCD the ß^s^ gene is inherited from both parents leading to homozygous HbSS (sickle cell anaemia) which is the most severe form (Ware *et al*., 2017). Recurrent SCD can have a detrimental effect on people’s quality of life, impacting educational attainment, employment status, psychosocial development and leading to recurrent hospitalization (Amaeshi *et al*., 2022).

A systematic literature review analysing data between 1980 and 2017 estimated global birth prevalence for homozygous SCD at 112 per 100 000 live births (95% CI, 101–123), with a birth prevalence of 1125 per 100 000 (95% CI, 680.43–1570.54) in Africa compared to 43.12 per 100 000 (95% CI, 30.31–55.92) in Europe (Wastnedge *et al*., 2018). Higher mortality rates in children under age 5 are also concentrated in Africa with 7.3 per 100 years (95% CI, 4.03–10.57) compared to 0.11 (95% CI, −0.24–0.46) in Europe (Wastnedge *et al*., 2018). Approximately 300,000 children are born yearly with SCD in the world, with sub-Saharan Africa (SSA) accounting for 75% of global cases (Salim *et al.*, 2021). Prevalence estimates of SCD in SSA countries are sensitive to accurate recording of births and diagnosis (Makani *et al*., 2013). Prevalence estimates of sickle cell trait for Cameroon, Democratic Republic of Congo (DRC), Gabon, Ghana and Nigeria are between 20% and 30% but in Uganda are estimated up to 45% (Regional Committee for Africa, 2006). In Angola, the prevalence of SCD is about 2%, with sickle cell carriers reaching 21% of the population (Brito *et al*., 2022). Recent estimates for Tanzania found a disease prevalence of 1.2% and trait prevalence of 20.3% using blood spot samples from children born to mothers with human immunodeficiency virus (Ambrose *et al*., 2020), however this is likely to be an underestimate due to sub-optimal screening.

In Africa 50–80% of those born with SCD die before the age of 5 which is in contrast to high income countries such as the United States of America where the survival rate for children under 5 years is 94% (Aygun and Odame, 2012). The population of newborns with SCD is increasing, a large fraction of SCD-related deaths in Africa occur prior to diagnosis, due to the absence of large scale newborn screening programs thus emphasizing a critical need to increase SCD awareness across SSA. Without screening and early intervention it has been estimated that between 50% and 90% of children die before their fifth birthday (Esoh *et al*., 2021). SCD also leads to substantial reduction in quality of life for adults and children (Kato *et al*., 2018).

Research undertaken in Nigeria reported mortality of up to 90% in people with SCD, however, recent estimates show that mortality rate is reducing and is likely to decrease to 50% in 20 years time due to increasing healthcare strategy and relative awareness (Makani *et al*., 2013). As part of the healthcare strategy in African countries to decrease morbidity and mortality, the Sickle Cell Program worked with the Ministry of Health to develop recommendations for standard of care delivery to SCD patients. These recommendations were incorporated into the national strategy for non-communicable disease (NCD) which was in keeping with the world health organization’s (WHO) strategy for NCD in the Africa (Makani, 2020). The recommendations included providing a health monitoring booklet for SCD patients and their family, new guidelines for managing SCD at primary and tertiary level, and the use of hydroxyurea, which reduces pain frequency and blood transfusion rate (Makani, 2020).

The WHO declared SCD a global health priority in 2006, and subsequently recommended the development of a public health strategy in Africa to reduce the SCD burden and mortality (Diallo and Guindo, 2014). As part of this strategy June 19th was designated a Sickle Cell Awareness Day and is observed annually to increase public knowledge and understanding of SCD (Thein and Thein, 2016). Effective health information seeking can lead to health-improving activities and serve as an essential tool for understanding how, why and when people access public health-related information (Fang *et al*., 2021; Shepherd *et al*., 2023). However, measuring the impact and benefit of disease awareness days remains a challenge (Tegegne *et al*., 2022). Typical epidemiological studies rely on large scale data collection, are costly, resource-intensive and have a limited geographical scope (Cao *et al*., 2021) and are particularly challenging to operationalize in low resource settings. An alternative real-time and low-cost method is therefore needed to monitor public awareness of a disease before, during and after the awareness day. Due to the wide coverage, rapid updating, low cost and anonymity of internet data, several studies have used internet search data, such as that provided by Google Trends (GT) to evaluate the potential impact of disease awareness days on online health information seeking behaviour OHISB (Havelka *et al*., 2020; Ajbar *et al*., 2021; Cao *et al*., 2021; Tegegne *et al*., 2022). Google Trends is a free, publicly available online tool that analyses searches conducted in Google Search and provides information tailored to geographical location and time using user-specified terms and restrictions (Google, 2009).

The increase in digital health innovations in recent years and increase in internet usage, particularly amongst younger populations in sub-Saharan Africa and the concentrated burden of SCD in this region provides an important research setting to explore online health information seeking behaviour (Holst *et al*., 2020). The aim of the project was to evaluate the impact of the World SCD awareness day in counties where SCD is a pervasive public health challenge using GT as a ‘surrogate’ of disease awareness.

## METHODS

GT is free and provides access to an unfiltered sample of real-time search requests made to Google. Data is anonymized, categorized and aggregated to display search interest in a particular topic from anywhere around the world. Each sampled data point is then scaled to the maximum number of searches over the selected location and time. This ‘relative popularity’ is presented as a Relative Search Volume (RSV) with values between 0 and 100 (Google, 2009). The RSV data are presented as a search volume index graph.

### Documentation of Google Trends use

**Fig. 1:**
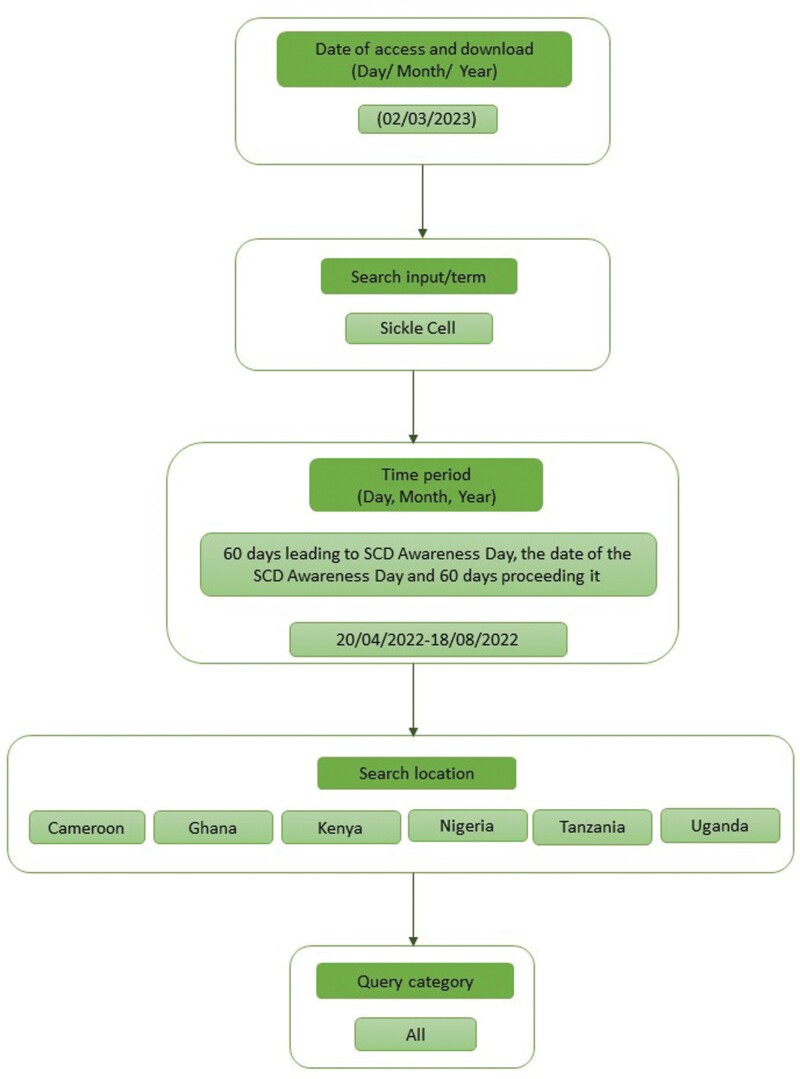
Visual schema of our GT search strategy.

To ensure transparency and the reproducibility of GT-based research, the checklist recommended by Nuti *et al.* was adhered to including; documenting rationale for search strategy, provide date(s) when GT was accessed and data downloaded, identify the query category used and provide the full search input(s) that were queried for using GT (Nuti *et al*., 2014). A flowchart depicting this process is presented in Figure 1.

### Search input

The term ‘sickle cell’ was used as the input for GT and ‘search term’ opted for instead of ‘topic’ for the search style. This was to ensure maximum inclusivity of sickle cell related searches. The category filter was set to ‘all’. Quotations were used to ensure unrelated phrases containing the word ‘sickle’, or ‘cell’, solely, were not returned in the searches. Countries were initially selected according to where the Sickle Cell Awareness Day takes place in Africa. Inclusion criteria for countries analysed was based on the high prevalence of sickle cell trait, mortality or disease burden associated with SCD, areas where the annual SCD awareness day was active and where sufficient GT data was available.

Nigeria, Ghana, and DRC have a sickle cell trait prevalence between 20% and 30% while some parts of Uganda have a prevalence as high as 45% (Regional Committee for Africa, 2006). Angola has a SCD prevalence rate of approximately 2% (Brito *et al*., 2022). Gabon has an estimated SCD prevalence rate of 1.8% (Minto’o *et al*., 2022) and Cameroon has an estimated prevalence rate of 0.6% (Alima Yanda *et al*., 2017). There is no national prevalence documentation of SCD in Tanzania, however an estimate of about 11,000 births annually has been recorded (Makani *et al.*, 2018) Western parts of Kenya have an estimated 4.5% of children born with SCD, and 18% of children born with sickle cell trait (Wanjiku *et al*., 2019). Angola, DRC, and Gabon could not be included in the analysis due to insufficient GT data.

### Search variables

Search conditions included data from 60 days preceding the Sickle Cell Awareness Day (19 June 2022), the awareness day itself, and up to 60 days post event, giving a total of 121 days. This was to ensure that data captured was related to OHISB prior to the awareness day and to observe any subsequent short-term changes to the trend. About 60 days was selected to ensure the RSV window was more appropriate for a 3 joinpoint mode as per previous work (Havelka *et al*., 2020; Ajbar *et al*., 2021).

### Data analysis

Data from the Google Trends explore page was retrieved in.csv format and opened in Microsoft excel (Microsoft Inc, Seattle WA, USA) on 2 March 2023. The independent variable was the day ID, where the 61st of the 121 days represented the Sickle Cell Awareness Day and the dependent variable was set to be the RSV value.

### Joinpoint analysis

A time trend analysis was carried out on the Google Trends data to access change in RSV as an indicator of OHISB leading up to and following the Sickle Cell Awareness Day (World Bank, 2023). A joinpoint regression analysis was used to identify points where a statistically significant change in trends occurs in the RSV data. The model assumed that the errors have a constant variance, and analysis was pre-set with the criteria to find a minimum of 0 and maximum of 3 joinpoints (Havelka *et al*., 2020; Ajbar *et al*., 2021). This was to capture an initial increase in RSV when the Sickle Cell Awareness Day takes place, a second for when trends may downturn after the Sickle Cell Awareness Day, and a third for when the downturn resumes back to the pre-Sickle Cell Awareness Day RSV. The model selection method was a permutation test, testing for an overall significance level at 0.05.

### RSV comparison before and after the Sickle Cell Awareness Day

To measure a difference in RSV before and after the Sickle Cell Awareness Day, the mean RSV in the 60 days before and the 60 days after the Sickle Cell Awareness Day were calculated. A paired, one tailed, *t*-test with a significance level of 0.05 was conducted to test for significant differences between these 2 means. This additional analysis was conducted to compliment the joinpoint analysis to explore if there was a significant and sustained change in RSV following the awareness day.

## RESULTS

### Cameroon

**Fig. 2:**
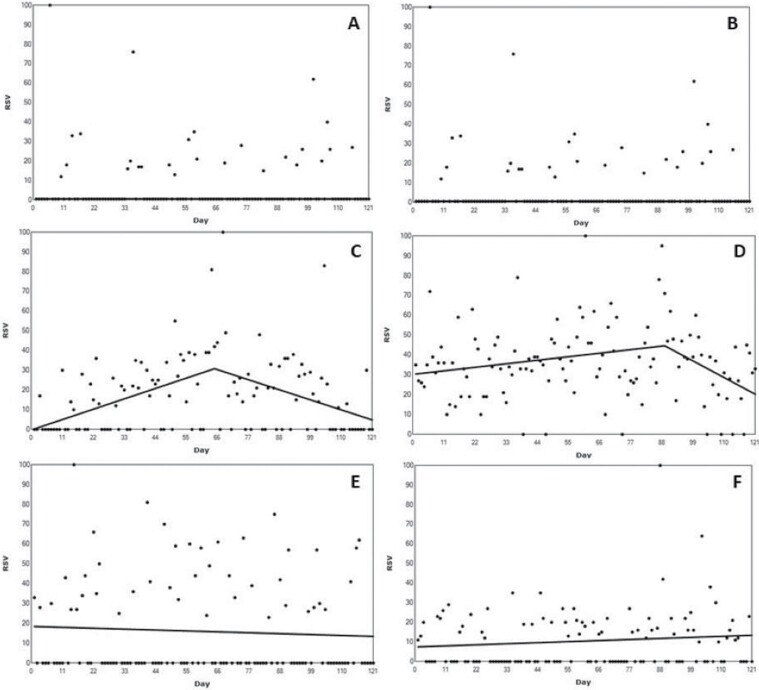
Changes in Relative Search Volume (RSV) for Sickle Cell Disease across the studied African countries. Each data point indicates the RSV measured on the specified day. RSV is the query share of a particular term for a given location and time period, normalized by the highest query share of that search term. Number of slopes present depends on the number of joinpoints identified. Joinpoints mark a statistically significant change in the linear slope of the trend in the studied time period. (A) RSV data for Cameroon. (B) RSV data for Ghana. (C) RSV data for Kenya. (D) RSV data for Nigeria. (E) RSV data for Tanzania. (F) RSV data for Uganda.

The RSV data for Cameroon is presented in Figure 2A. No significant joinpoints were found during analysis of the Cameroon RSV data (*p* = .56). The result of the before (*M* = 7.81, SD = 18.21) and the after (*M* = 5.14, SD = 12.14) RSV comparison revealed no significant difference, *t* (58) = 0.88, *p* = .83).

### Ghana

RSV data for Ghana is presented in Figure 2B. No significant joinpoints were found in the analysis (*p* = .75). The comparison between RSV data before (*M* = 28.25, SD = 23.36) and after (27.34, SD = 26.04) the awareness day also revealed no significant difference, *t* (58) = 0.20, *p* = .85).

### Kenya

Analysis revealed a significant one-joinpoint model (*p* < .001) at day 65, following an approximate 300% increase in RSV following the awareness day at day 61, before RSV data returned to previously observed levels. The comparison of RSV data before (*M* = 13.86, SD = 14.79) and after (19.90, SD = 22.25) day 61, revealed no significant difference, *t* (58) = 1.55, *p* = .13. Data for the Kenya RSV data are presented in Figure 2C.

### Nigeria

Analysis revealed a significant one-joinpoint model (*p* = .03) on day 89, despite the maximum RSV (100) on the day of the campaign (day 61), representing a 100% increase in RSV. The difference in RSV from before (*M* = 34.58, SD = 15.62) the campaign to after (*M* = 37.32, SD = 21.24) was not significant, *t* (58), = 0.79, *p* = .43. RSV data for Nigeria are presented in Figure 2D.

### Tanzania

The RSV data for Tanzania are presented in Figure 2E. No significant joinpoints were found for the RSV data (*p* = .51). The result of the before (*M* = 17.00, SD = 25.17) and the after (*M* = 14.71, SD = 22.62) RSV comparison revealed no significant difference, *t* (58) = 0.59, *p* = .56).

### Uganda

The RSV data for Uganda are presented in Figure 2F. No significant joinpoints were found in the Uganda RSV data (*p* = .20). However, the increases in RSV from before (*M* = 7.54, SD = 10.84) to after the campaign (*M* = 15.86 = 5, SD = 23.25) was statistically significant, *t* (58) = 2.15, *p* = .04.

## DISCUSSION

This study explored the impact of the African Sickle Cell Awareness Day on OHISB across several African countries using GT data. In Cameroon and Tanzania, there were no significant changes in OHISB observed as a result of the Sickle Cell Awareness Day. In Ghana, although there was no significant change in data trends around the Sickle Cell Awareness Day, the RSV data remained relatively high from across the study period. In Kenya and Nigeria, there were significant increases in RSV following the Sickle Cell Awareness Day. In Kenya, there was an immediate increase in RSV following the Sickle Cell Awareness Day (day 65), which then returned to previously observed levels. This suggests that the Sickle Cell Awareness Day may have raised awareness about SCD, but the impact in terms of OHISB was not sustained. In Nigeria, there was a significant increase in RSV after the Sickle Cell Awareness Day (day 89), although the maximum RSV was on the Sickle Cell Awareness Day itself (day 61). This suggests that the Sickle Cell Awareness Day may have had some impact on OHISB, but again, the effect was not sustained. In Uganda, there was a statistically significant increase in RSV from before to after the Sickle Cell Awareness Day, but no significant joinpoints. This suggests that the Sickle Cell Awareness Day may have had a delayed impact on OHISB in Uganda. Overall, the results of the study suggest that the impact of the Sickle Cell Awareness Day in Africa varies across different countries and generally the impact is short lived.

In African countries such as Uganda and Ghana, cross-sectional studies conducted to evaluate people’s understanding and knowledge about SCD, revealed that despite the level of awareness, their knowledge about the disease was insufficient (Tusuubira *et al*., 2018; *Brown et al*., 2022). Therefore, continuous and more effective efforts are required to promote sickle cell awareness within communities such as improved education in health and educational settings such as schools, concentrated efforts in rural communities where traditional and complementary medicine is more likely to be practiced, improved strategies to increase screening uptake and campaigns focusing on de-stigmatizing SCD (Taiwo *et al*., 2011; Tusuubira *et al*., 2018; Brown *et al*., 2022). Although GT has been utilized in evaluating the impact of Global Health Awareness days on OHISB in other regions such as the Arabian Peninsula and Central and South America (Havelka *et al.*, 2020; Ajbar *et al*., 2021), this study represents the first of its kind to apply GT to assess the impact of the Sickle Cell Awareness Day in African Countries on OHISB. Similar to our previous research, both studies demonstrated that the influence of Global Health Awareness Days on OHISB varies among countries and lacks consistency. Despite using shorter search periods of 36 days and 35 days respectively, the studies by Ajbar *et al.* and Havelka *et al.* showed more significant trend data in more countries compared to the current study, which employed a more extended search period of 121 days for more impactful observations. This is probably due to higher levels of internet penetrance in the Arab countries researched (Simsim, 2011) and higher interest among the Arab world in seeking online health information (Bahkali *et al*., 2016). Internet penetrance for countries in this study ranged from 24.6% (in Uganda) to 68.2% (in Ghana) (Kemp, 2023a,c).

In Kenya and Nigeria the impact of the observed findings reported here is consistent with previous research that has shown that disease awareness days can lead to a significant and steep spike on OHISB, but that this spike is relatively short lived (Havelka *et al*., 2020; *Ajbar et al.*, 2021). This pattern can be attributed to a number of factors. Firstly, GT only accounts for one element of online health information, with other searching taking place via other search engines and platforms including social media. Mahabir *et al.* introduced a framework called stimulus-awareness-activism, which proposes that an individual’s awareness about a certain topic leads to increased online and offline activities related to that topic (Mahabir *et al*., 2018). In a similar vein, Mahroum *et al.* examined digital behaviours in response to a Chikungunya outbreak by analyzing the interaction between various data sources like website searches and social networks (Mahroum *et al*., 2018). They discovered that Google Trends had a positive impact on Twitter activity. Essentially, users tended to search for ‘Chikungunya’ on Google when notified about the cases, and then engaged with the topic on Twitter.

Moreover, browsing specific websites related to the topic can enhance awareness. Users often discover these websites through Google searches and subsequently use them as a source of health information. For example, Kranenburg *et al.* analysed Google Trends data for ‘blood donation’ in relation to World Blood Donor Day, reporting that national-level blood bank websites received double the usual number of visits, and this correlated positively with the Google RSV for ‘blood donation’. Consequently, it is possible that the initial surge in seeking online health information lasts longer than the available data suggests, as it might be manifested through other data streams. It is recommended that future studies explore the connections between website traffic to disease-specific sources and relevant Google RSVs (Shepherd *et al*., 2023).

The low levels of internet penetrance in some sub-Saharan African countries means the impact of health awareness campaigns on OHISB may be limited. Particularly in those countries where no significant trends in online health information seeking were found and RSV was largely unchanged; Cameroon, Tanzania and Ghana. A systematic review looking at the effectiveness of health awareness days across a range of diseases mostly including cancer and a range of countries including the UK, USA and globally, noted an increase in activity around the time of the campaign but conflicting results on whether this improved health outcomes or influenced health behaviour (Vernon *et al*., 2021). Few studies have described how people from sub-Saharan African countries seek health information, however a qualitative study of older adults with low income from Ghana described that most study participants sought health information through healthcare practitioners, media including TV and radio, family and friends. There was no mention of active online health information seeking. This highlights the importance of using multiple methods to disseminate health-related information (e.g. for SCD, covering pre- and post-natal screening, importance of early diagnosis, early identification of disease complications and management options), including using existing infrastructures and traditional methods (e.g. local healthcare providers, newspapers and radio) and not relying on online information seeking, particularly with the variation in internet penetrance across sub-­Saharan Africa (World Bank, 2023). More research is required to assess the impact and benefit of disease awareness days in SSA, in particular determining the best methods by which this can be achieved and also the impact of traditional methods of health information sharing and whether this leads to improved health outcomes. Language and health literacy has also been noted as a barrier to older adults acquiring health information (Agyemang-Duah *et al*., 2020).

### Limitations and strengths

There are limitations with using internet search query data and Google Trends data specifically as a measure of OHISB. Firstly, only those with internet access can be accounted for in OHISB data. Therefore, our findings are only valid for health information seeking that takes place online. Those without internet access may engage in health information seeking behaviour through alternative means, such as contacting a health professional, watching news, listening to radio, and attending health seminars. However, the percentage of internet users was relatively high in most of the studied countries, only in DRC (22.9%) the internet penetration was substantially lower compared to that in the other countries and thus could explain why we had no GT data for analysis in the country (Kemp, 2023b). But surprisingly, Gabon and Angola with internet penetrance of 60% and 36% internet penetrance respectively did not have enough GT data for analysis (World Bank, 2023). Secondly, the observed interest level is limited to those who use Google as a search engine. However, in the studied time period Google represented 70% of the search engine market share in Africa (Statscounter, 2023). These points indicate a sufficient level of internet access and use of Google as search engine in the African countries to use Google Trends as a proxy for OHISB. The over interpretation of trends is discussed as a limitation of using GT for research. The calculation of the search value index (RSV) is dependent on mathematical assumptions and approximations, which are not public and may obscure true trends in search traffic. However, previous evidence suggests trends have been accurate in approximating the seasonality of conditions (Seifter *et al*., 2010) and at predicting influenza outbreaks comparable to the US Centres for Disease Control health surveillance mechanisms (Carneiro and Mylonakis, 2009). A systematic review on the use of Google Trends in health-related research revealed poor documentation of the methodology in most studies, limiting reproducibility of study findings (Nuti *et al.*, 2014). We have adhered to their documentation recommendations to ensure transparency and reproducibility of our methodology allowing for potential comparisons of findings over time.

## CONCLUSION

Overall, the study provided valuable insights into the effectiveness of the Sickle Cell Awareness Day and its influence on OHISB in sub-Saharan Africa. The results of the study suggest that the impact of the Sickle Cell Awareness Day on OHISB in African countries varies across different countries and it may be less significant and inconsistent despite internet usage by people in these countries. The failure of the Sickle Cell Awareness Day to consistently influence OHISB in a time of high and constantly increasing internet use potentially calls into question the effectiveness of the awareness day itself. This finding may also highlight inconsistent internet access and use of other self-education modalities in the region. Our findings suggest more targeted campaigns using different methods of information dissemination may be required to improve public awareness of Sickle Cell Disease and influence OHISB. Additionally, this being the first study to use GT in evaluating the impact of the Sickle Cell Awareness Day on OHISB in African countries, the study highlighted the limitations of using GT data for analysis in some African countries due to insufficient data. Future research could explore other sources of internet data or conduct surveys to gain a more comprehensive understanding of the impact of the Sickle Cell Awareness Day on OHISB in African countries. Nonetheless, this study has provided a starting point for understanding the impact of the Sickle Cell Awareness Day on OHISB and highlights the need for tailored approaches to health communication in African countries.
